# Formyl peptide receptor type 2 agonists to kick‐start resolution pharmacology

**DOI:** 10.1111/bph.15212

**Published:** 2020-09-20

**Authors:** Mauro Perretti, Catherine Godson

**Affiliations:** ^1^ The William Harvey Research Institute, Barts and The London School of Medicine Queen Mary University of London London UK; ^2^ Centre for Inflammation and Therapeutic Innovation Queen Mary University of London London UK; ^3^ Diabetes Complications Research Centre, Conway Institute & School of Medicine University College Dublin Dublin Ireland

**Keywords:** annexin A1, GPCRs, lipoxins, small molecule agonists, therapeutic innovation

## Abstract

One way to develop innovative approaches for the treatment of chronic diseases is to exploit the biology of the resolution of inflammation. With this terminology, we identify the integrated and complex network of mediators and pathways that ensure a timely and spatially regulated inflammatory response. Pro‐resolving mediators act on specific receptors. This provides an opportunity for developing a new arm of pharmacology we have termed “resolution pharmacology.” Here we present the reasoning behind the need to develop new medicines based on resolution and use a prototype GPCR as an example. Understanding how the formyl peptide receptor type 2 (FPR2) operates in a cell‐specific manner can guide the development of agonists as new therapeutics that could be of benefit as a therapy or co‐therapy for several diseases that affect our society. FPR2 agonists would be among the first drugs to establish “resolution pharmacology” as the pharmacological approach for the third decade of the millennium.

**Abbreviations**
ALX: lipoxin A_4_ receptor
AnxA1: annexin A1
COVID‐19: coronavirus disease 2019
FPR: formyl peptide receptor
FPR2: formyl peptide receptor type 2
TM: transmembrane

## 1 INTRODUCTION

The formyl peptide receptor type 2 (FPR2) is a member of the formyl peptide receptor (FPR) family. The human FPR family is clustered on chromosome 19 and encodes a family of three Class A GPCRs (Ye et al., 2009). This class of receptors is characterised by a short N‐terminal region, an NPXXY motif in transmembrane (TM) region 7 and an E/DRY motif that bridges TM3 and TM6 stabilising inactive receptor conformations. FPRs play a critical role in innate immune responses through recognition of pathogen‐associated molecular patterns (PAMPs) and damage‐associated molecular patterns (DAMPs). FPR2 is highly expressed in myeloid cells and also in cells of diverse origin including endothelia, epithelia, smooth muscle cells, chondrocytes and fibroblasts.

FPR2 displays many intriguing physiological and pharmacological responses as reflected, initially, by its activation by diverse ligands including proteins, peptides and lipids (Perretti et al., 2002). Thus, FPR2 is referred to as ALX, an acronym for the lipoxin A_4_ (LXA_4_) receptor and, indeed, the terminology ALX/FPR2 or FPR2/ALX should be used to identify it when activated by lipid or protein agonists, respectively (Ye et al., 2009). Intriguingly, FPR2 also conveys pro‐inflammatory effects in response to specific ligands, like the acute‐phase protein serum amyloid A. It is plausible that ligand‐specific receptor conformational changes associated with downstream signalling are the main reason for the divergent properties reported in the literature (e.g. T. Chen et al., 2020). Similarly, a degree of versatility has been reported with respect to signalling pathways: classically associated to an inhibitory G protein, FPR2 signalling provokes formation of IP_3_ and calcium fluxes together with an involvement of MAPKs. It is now evident that it can engage distinct G proteins and hence activates different signalling cascades, presumably in a target cell‐specific manner (Inoue et al., 2019). Congruently, FPR2‐depedent cAMP activation, presumably following engagement with a G protein containing a G_s_ alpha subunit, was observed following addition of the agonist aspirin‐triggered resolvin D1 (Mottola, Chatterjee, Wu, Chen, & Conte, 2017). Our own work identified FPR2 as the receptor for annexin A1 (AnxA1), an anti‐inflammatory protein and the same receptor engaged by LXA_4_, which at the time was identified a prototypic anti‐inflammatory endogenous mediator (Perretti et al., 2002). In line with the degree of ligand specificity indicated above, for some time, the genuine nature of FPR2 has remained unclear, with data indicating pro‐inflammatory properties following receptor activation and other studies showing its anti‐inflammatory activities upon activation on target cells and tissues (K. Chen et al., 2010; X. Chen et al., 2019; Dufton et al., 2010; Liang et al., 2020). We now know that the nature of the responses downstream of FPR2 activation is more complex, yet the receptor could be genuinely indicated as a *master switch in promoting the resolution of inflammation.*

## 2 RESOLUTION OF INFLAMMATION AND RESOLUTION PHARMACOLOGY

The resolution of inflammation reflects the integration of biological processes that are operative in a protective and physiological inflammatory response, where the onset‐to‐peak phase of the host response to injury and infection is followed by temporally appropriate and controlled resolution, leading to the restoration of normal tissue/organ function. In the context of pathological inflammation, which is often chronic, or transient and relapsing, a low‐grade inflammation occurs. This ongoing low degree of inflammation is thought to fail to set in motion the integrated processes of resolution (Serhan & Savill, 2005). Therefore, it is of significant importance to identify processes and mediators that are central to promote an active resolution. As an example, incubation of human and mouse macrophages with TNF‐α induces FPR2 expression through *de novo* synthesis (Gobbetti et al., 2014). Mediators, targets and processes of resolution have been described in several excellent reviews and will not be reviewed here (de Gaetano et al., 2018; Perretti, Cooper, Dalli, & Norling, 2017; Serhan, 2014). What is important though is that FPR2 is able to promote several, if not all of the processes that characterise the resolution of inflammation biology, including blocking neutrophil extravasation, promoting non‐phlogistic monocyte recruitment, inducing neutrophil apoptosis, enhancing macrophage phagocytosis as well as macrophage efferocytosis, altering macrophage phenotype and, as emerging more recently, instructing stromal cells to favour repair (Perretti et al., 2017). We propose that in settings of chronic disease, FPR2 might be able to reinstruct immune and tissue cells to favour, if not the regaining of homeostasis a more stable status of allostasis. Promoting resolution addresses a key unmet need given the challenges of insidious chronic inflammation that underpin numerous prevalent diseases. The challenge is to harness FPR2 receptor activation towards developing novel medicines.

**FIGURE 1:**
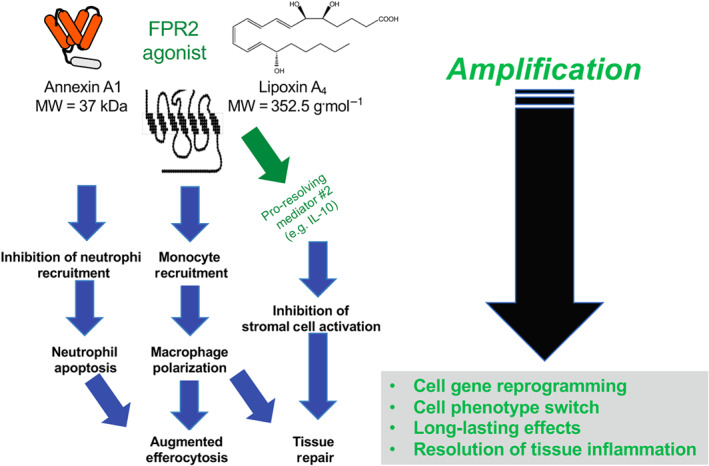
Schematic representation of the integrated actions of pro‐resolving mediators. Left panel: Annexin A1 and lipoxin A_4_ are presented as exemplar ligands for formyl peptide receptor type 2 (FPR2), the pro‐resolving receptor. By activating this GPCR, the two agonists promote the integrated bioactions of resolution for a general reorganisation of the affected tissue or organ, to enable termination of inflammation and regain of whole or partial tissue/organ functionality. Right panel, another distinctive element of pro‐resolving therapeutics lies in the fact that their agonistic activity can amplify their effects (see interwoven network of actions of the left) and promote reprogramming of target cells. This would ensure (a) efficacy even when the agonist is no longer there, (b) a switch in cell phenotype or polarisation and (c) all‐in‐all longer‐lasting pharmacological efficacy. These characteristics require consideration for drug development with respect to pharmacokinetics, administration route and frequency

A few years ago, we have defined “resolution pharmacology” as a new branch that will identify drugs developed on pro‐resolving mediators and acting at pro‐resolving receptors (Perretti, Leroy, Bland, & Montero‐Melendez, 2015). Important here is the new approach to drug discovery for inflammatory diseases, since these new drugs ought to be agonists and hence activate their receptor(s) and promote a gene reprogramming that would change the phenotype of target cells. This represents a 180° shift in the way one would treat chronic diseases, promoting reparative processes instead of blocking a given mediator or pathway (the latter is the approach taken over the last 100 years, with receptor antagonists, enzymatic inhibitors, anti‐cytokine biologics, etc.). Figure 1 presents this prediction in a schematic way. First, activation of the pro‐resolving receptor like FPR2 by its agonists (e.g. two endogenously generated agonists indicated here, AnxA1 and LXA_4_) sets in motion the *integrated actions* of resolution. This cascade could involve also expression and/or release of other pro‐resolving mediators so that a reparative network of resolution is then displayed in the target tissue. A relevant example here is the release of IL‐10 induced by AnxA1 and LXA_4_ as shown in the context of gut inflammation (Souza et al., 2007) or liver fibrosis (Locatelli et al., 2014). The latter study revealed another circuit of resolution, whereby LXA_4_ induced AnxA1 expression as an endogenous pathway to attenuate liver and kidney diseases in dysmetabolic settings of obesity (Börgeson et al., 2015). Moreover, agents that elevate intracellular cAMP can promote the synthesis of AnxA1 with a positive effect on the resolution of experimental pleural inflammation (Lima et al., 2017). Figure 1 illustrates another unique value of resolution biology, the possibility to re‐programme target cells to enable long‐lasting reparative and regenerative effects in the tissue of interest. This is a central point for resolution pharmacology, to have a clinically beneficial effect on curbing ongoing disease. The therapy ought to change the activity and phenotype of target cells and ensure that the new subsumed status remains consistent for some time.

Agonism at a pro‐resolving receptor provides this unique opportunity with the potential to be a game changer in the context of chronic diseases characterised by ongoing inflammatory processes. The immune modulation induced by FPR2 activation includes attenuation of signals coupled to IL‐1β generation and downstream targets including IL‐6 (Brennan, Mohan, McClelland, Tikellis, et al., 2018). These responses may be amenable to mimicry in the context of the unmet need presented by the cytokine storm of coronavirus disease 2019 (COVID‐19).

## 3 FPR2 AS A PROTOTYPE TO KICK‐START RESOLUTION PHARMACOLOGY

An important cue now is how can one predict the pharmacological properties on new molecules developed for agonism at a given pro‐resolving receptor? We have recently reported that AnxA1 interacts with FPR2 to activate a specific signalling cascade that leads to activation of AMPK (McArthur et al., 2020). The ability of AMPK signalling to direct macrophage polarisation in the context of muscle injury repair was known (Mounier et al., 2013); however, the inciting stimulus upstream was not. We could demonstrate that FPR2 was the main checkpoint here, with the agonist AnxA1 being provided by infiltrating neutrophils. The ultimate outcome of the AnxA1/FPR2/AMPK pathway resulted in the occurrence of a proper inflammatory reaction after muscle injury, with recruitment of macrophages to the site of injury and their polarisation towards an anti‐inflammatory tissue‐reparative phenotype that ensured timely tissue repair and muscle regeneration. Mice lacking AnxA1 showed impaired muscle repair after experimental injury (McArthur et al., 2020). This study indicates that assessment of AMPK phosphorylation downstream application of FPR2 agonists (AnxA1 mimetics, LXA_4_ analogues or new chemical entities) could help identify early whether an agonist at this receptor would be able to polarise macrophages and promote tissue‐reparative processes.

On a similar vein, we have demonstrated that LXA_4_ can drive macrophage polarisation. In the context of experimental obesity, LXA_4_ attenuated the increased M1:M2 ratio observed in adipose, TNF‐α generation and downstream liver and kidney inflammation (Börgeson et al., 2015). LXA_4_ treatment was associated with increased AnxA1. Activation of AMPK has been shown to result in equivalent responses of adipose liver and kidney in this system (Börgeson et al., 2017). In mouse models of diabetes, LXA_4_ induced regression of atherosclerosis and renal fibrosis. Altered gene expression patterns induced by LXA_4_ treatment in aorta (Brennan, Mohan, McClelland, de Gaetano, et al., 2018) and kidney (Brennan, Mohan, McClelland, Tikellis, et al., 2018) were consistent with a switch in phenotype of infiltrating macrophages.

An interesting aspect emerged from the AMPK study, the fact that FPR2 expression was markedly attenuated if not totally absent from macrophages that had acquired a tissue‐reparative mode. This was evident both at the mouse level (with immunohistochemistry in the damaged muscle and quantification of gene expression product in sorted cells) and in human macrophages (McArthur et al., 2020). In the latter case, cells lost FPR2 cell surface expression when polarised with IL‐4. These data indicate the possible existence of a fail‐safe mechanism whereby the AnxA1/FPR2/AMPK pathway cannot operate in cells that have already switched their phenotype towards a pro‐resolving one. We hypothesise that such a mechanism will prevent an overshooting of the pro‐resolving signal and as such will avoid risks of excessive dampening of inflammation. In sepsis, LXA_4_ decreases systemic inflammation without compromising host defence and can reduce pathogen virulence by inhibiting quorum sensing (Wu et al., 2016). Investigation of single‐nucleotide polymorphisms in the FPR2 gene identified that the rs11666254 polymorphism was associated with decreased FPR2 mRNA and protein expression, molecular changes that are functionally associated with susceptibility to sepsis after traumatic injury (Zhang et al., 2017).

## 4 RESOLUTION PHARMACOLOGY FROM FPR2 BIOLOGY

**FIGURE 2:**
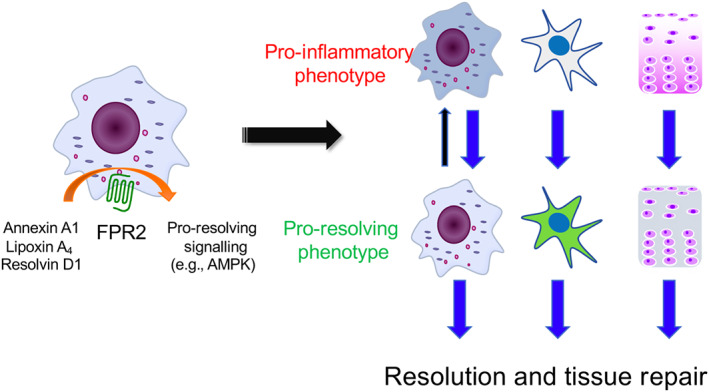
Pro‐resolving receptor agonism to modify the phenotype of target cells. Our recent work has identified AMPK as the signalling determinant downstream of formyl peptide receptor type 2 (FPR2) activation to switch the phenotype of muscle macrophages from a pro‐inflammatory to a pro‐resolving reparative one (McArthur et al., 2020). This dataset prompts the hypothesis that definition of the signalling pathways is of importance to accelerate the development of pro‐resolving drugs, with the caveat that the relevant pathway may be cell and disease specific. Here we depict macrophage FPR2 that responds to its agonists leading to macrophage switch. Moreover, the same need to switch the phenotype of cells like fibroblasts and chondrocytes should prompt identification of the predictive signalling pathway conducive to the wanted outcome, for example, not activating fibroblasts to myofibroblasts or anabolic chondrocytes

We consider FPR2 the most intriguing pro‐resolving receptor by far. Mice lacking the receptor orthologues display often significant phenotypes in settings of experimental diseases. We propose that to successfully exploit the biology of resolution to guide the development of novel molecules, one has to identify pro‐resolving signalling cascades that are conducive to the desirable pro‐resolving actions. Early implementation of the appropriate predictive signal(s) at the level of preclinical development will increase the chances of success for molecules that will make it to clinical trial. As a consequence, definition and validation of the pro‐resolving signalling readout must be defined in the relevant target cells and applied early on in screening programmes. Above, we have discussed exemplars of signalling pathways relevant to macrophage phenotype switch muscle injury repair, regression of atherosclerosis and renal fibrosis. It is plausible that the relevant pro‐resolving signalling would be to some degree specific for a given target cells, in a given disease setting. Figure 2 depicts this observation with the example of macrophages and the need to apply similar approaches to chondrocytes (if the endpoint is to develop cartilage‐regenerating therapies), fibroblasts (for anti‐fibrotic approaches), or other cell targets (e.g. alveolar macrophages for lung allergy or gut epithelium for inflammatory bowel disease).

FPR2 agonists are being developed and tested also in man. Indeed, the interesting biology of the receptor has stimulated a plethora of medicinal chemistry programmes, some reported in recent reviews (Corminboeuf & Leroy, 2015; Perretti et al., 2015). The recent resolution of the receptor in agonist‐binding mode would certainly favour further developments (T. Chen et al., 2020). Meanwhile, synthetic lipoxin mimetics, which are FPR2 agonists, have been generated and investigated in preclinical settings (de Gaetano et al., 2019). A Phase I trial has been conducted with a small‐molecule FPR2 agonist developed by Actelion, compound ACT‐389949 (Stalder et al., 2017). More recently, Bristol‐Meyers Squibb has concluded a Phase I trial for a different small‐molecule selective FPR2 agonist, compound BMS‐986235 (ClinicalTrials.gov Identifier NCT03335553). The medicinal chemistry underpinning the development of this compound has recently been published (Asahina et al., 2020) with the report of its tissue protective properties in experimental heart failure. These recent developments represent an exciting phase for determining the effective potential that targeting FPR2 may offer for the development of new therapies. It is interesting to note how the most plausible application is for heart failure rather than pathologies classically identified as inflammatory. In preclinical settings, AnxA1 has been shown to impact positively on macrophage remodelling in the post‐infarct heart, through induction of a pro‐angiogenic macrophage phenotype (Ferraro et al., 2019). Studies in large animals with adenoviral delivery of AnxA1 confirmed this outcome. This work is consistent with the study of Qin et al. (2017) who have identified the heart‐protective properties of first‐generation FPR2 small‐molecule agonists (compound 43 and compound 17b) in acute myocardial infarct, proposing that a better outcome was associated with a biased agonism against intracellular calcium fluxes and in favour MAPK signalling cascade. More recently, García et al. (2019) reported the reparative actions of a dual FPR1/FPR2 agonist used as a proof‐of‐concept tool. Given orally over a 4‐week period, compound 43 afforded a high degree of protection in a model of heart failure, linking beneficial outcomes for the animals to its ability to polarise macrophages towards a type 2 phenotype. It is unclear how this cellular phenotype overlaps with the angiogenic macrophage promoted in the heart by AnxA1; in any case, there is at least a partial overlap between the properties of the synthetic and natural FPR2 agonists. We should note also that the beneficial responses to FPR2 activation in infarction have not been linked solely to tissue‐mediated effect, for example, centred on myocardial macrophages, but also to systemic responses, as shown by the ability of the prototype peptide agonist WKYMVM to mobilise angiogenic cells (Heo et al., 2017).

## 5 CONCLUSION

While this discussion has focused on heart failure since motivated by ongoing Phase II trials, we propose that FPR2 agonists can reap the benefits of the biology of resolution of inflammation to re‐programme relevant target cells in a variety of diseased tissues. Thus providing a fresh approach to the clinical management of several debilitating diseases beyond those primarily affecting the heart. Early characterisation of FPR2 post‐receptor signalling is required to incite the desirable biological properties and it will expedite the drug development process, while at the same time augmenting the chances of identifying molecules that will have the correct pharmacodynamic properties. This will increase the odds of efficacy in man, ultimately leading to patient benefit. There is a growing appreciation that differential responses to FPR2 activation may reflect agonist bias towards specific intracellular pathways and that, as such, pro‐resolving agonists may act as biased allosteric modulators as recently shown for LXA_4_ (Ge et al., 2020). FPR2 agonists may kick‐start the establishment of “resolution pharmacology” as a new branch of the discipline, typified by agonists that re‐programme target cells to dampen ongoing inflammatory processes to favour reparative and regenerative processes within the patients themselves.

### 5.1 Nomenclature of targets and ligands

Key protein targets and ligands in this article are hyperlinked to corresponding entries in http://www.guidetopharmacology.org, the common portal for data from the IUPHAR/BPS Guide to PHARMACOLOGY (Harding et al., 2018) and are permanently archived in the Concise Guide to PHARMACOLOGY 2019/20 (Alexander et al., 2019).

## CONFLICT OF INTEREST

M.P. is on the Scientific Advisory Board of ResoTher Pharma AS, which is interested in the development of AnxA1‐derived peptides for cardiovascular settings. M.P. consults for Bristol‐Meyers Squibb. C.G. is an inventor on patent application “Heterocyclic lipoxin analogues and uses thereof” that is currently under review (PCT/EP2017/070979).
